# Additive Manufacturing of Devices Used for Collection and Application of Cereal Rust Urediniospores

**DOI:** 10.3389/fpls.2019.00639

**Published:** 2019-05-15

**Authors:** Zacharias A. Pretorius, Gerrie J. Booysen, Willem H. P. Boshoff, Jozua H. Joubert, Gerrie J. Maree, Johan Els

**Affiliations:** ^1^Department of Plant Sciences, University of the Free State, Bloemfontein, South Africa; ^2^Centre for Rapid Prototyping and Manufacturing, Central University of Technology, Bloemfontein, South Africa; ^3^Product Development Technology Station, Central University of Technology, Bloemfontein, South Africa

**Keywords:** 3D printing, atomizer, cereal rust fungi, cyclone collector, spore collector, spore applicator, urediniospores

## Abstract

Optimized inoculation procedures are an important consideration in achieving repeatable plant infection when working with biotrophic rust fungi. Several plant pathology laboratories specializing in rust research employ a system where the collection and application of fungal spores are accomplished using an exchangeable gelatin capsule. Urediniospores are collected from erumpent pustules on plant surfaces into a capsule fitted to a cyclone collector controlled by a vacuum pump. By adding light mineral oil to the same capsule, the spore suspension is then sprayed onto plants by means of a dedicated atomizer (inoculator) connected to an air pressure source. Although devices are not commercially available, modern day technologies provide an opportunity to efficiently design and manufacture collectors and inoculators. Using a process called Additive Manufacturing (AM), also known as “3D printing,” the bodies of a collector and inoculator were digitally designed and then laser-sintered in nylon. Depending on availability, copper or aluminum tubes were fitted to the bodies of both devices afterward to either facilitate directed collection of spores from rust pustules on plant surfaces or act as a siphon tube to deliver the spore suspension contained in the capsule. No statistical differences were found between AM and metal inoculators for spray delivery time or spore deposition per unit area. In replicated collection and inoculation tests of wheat seedlings with urediniospore bulks or single pustule collections of *Puccinia triticina* and *P. graminis* f. sp. *tritici*, the causal organisms of leaf rust and stem rust, consistent and satisfactory infection levels were achieved. Immersing used devices in acetone for 60 s followed by a 2 h heat treatment at 75°C produced no contaminant infection in follow-up tests.

## Introduction

Several methods are available for controlled inoculation of cereal plants with urediniospores of rust fungi. In general, the method of choice depends on the number of plants to be inoculated, their growth stage, source and amount of inoculum, and available research infrastructure (Knott, 1989). Procedures include the application of dry spores to plant surfaces by dusting, either directly or by using a carrier such as talcum powder, brushing of plant surfaces with sporulating lesions on infected plants, transferring dry spores to leaf surfaces using a moistened spatula or similar instrument, dipping seedling leaves into water on which spores are floated, or by spraying plants with a spore suspension (Browder, 1971; Knott, 1989; Roelfs et al., 1992). Urediniospores could be suspended in water containing a surfactant, in isoparaffinic, light mineral oil products (Roelfs et al., 1992), e.g., Soltrol^®^ 130 (Chevron Phillips Chemical Company) or, more recently, in Novec^TM^ 7100 (3M^TM^) engineered fluid (Sørensen et al., 2016).

Browder (1965) described the construction of an air pressure operated brass atomizer delivering a spore-oil suspension from an inoculum reservoir in an attached glass vial. Browder (1971) developed a system to collect spores by means of a cyclone collector into a gelatin capsule (size 00) which could be directly transferred, after adding carrier oil, to an atomizer (also referred to as an inoculator). Although the design of atomizers used may differ, cereal rust facilities such as the University of Minnesota, ARS-USDA Cereal Disease Laboratory, Agriculture and Agri-Food Canada, CIMMYT, and laboratories in Australia and South Africa (Kolmer et al., 2011; Fetch et al., 2015; Pretorius et al., 2015; Terefe et al., 2016; Elmansour et al., 2017; Lan et al., 2017; Steffenson et al., 2017) have all reported the application of spores suspended in light mineral oil. If a supply of clean collectors and inoculators and a flushable inoculation booth are available, many isolates can be handled with ease. This approach is particularly suited to rust race analyses where several sets of differential lines need to be inoculated with different isolates in one session.

The devices described by Browder (1971) are not commercially available and are manufactured by a conventional milling process that requires a specialized workshop and skilled personnel. Likewise, costs, and the availability of a suitable carrier oil and gelatin capsules could be prohibitive in some countries. From experience we have also noted variation in delivery efficiency between batches of atomizers manufactured from metal. This often impacts negatively on experiments due to the over or under application of inoculum.

The objective of this project was to design a new collector and atomizer, using the basic operational principles proposed by Browder (1971), but employing Additive Manufacturing (AM) to facilitate a more consistent and easy to produce product. AM refers to a process by which a three-dimensional (3D) computer-aided design (CAD) is used to construct a component layer by layer by depositing material available in a fine powder form (Weller et al., 2015). The term “3D printing” is often informally used as a synonym for AM and allows the cost-efficient production of customized products (Weller et al., 2015). AM, however, is a more comprehensive description as it encompasses the entire range of technologies involved in efficiently translating model data into a physical object (Gibson et al., 2010). A variety of different metals, plastics and composite materials may be used in the manufacturing process (Levy et al., 2003). The technology has many applications, e.g., in aerospace, automotive, biomedical and engineering fields, and for manufacturing industrial machines and consumer products (Guo and Liu, 2013; Wohler et al., 2017). In addition, AM has evolved from rapid prototyping to construction of end-user products (Sossou et al., 2018).

## Materials and Methods

### Devices, CAD and 3D Printing

In a cereal rust context, the basic principle of the interchangeable device-capsule system is the controlled collection of fungal spores and expulsion of small amounts of inoculum in suspension, often in oil, onto plant parts. By connecting an air vacuum to the cyclone collector, fungal spores are drawn up through the copper intake tube and accumulate on the inside walls and/or in the conical bottom of the capsule. Once oil is added and the capsule fitted to an inoculator, air pressure applied to the inlet socket allows the spore suspension to escape through a siphon tube before being discharged as a fine mist.

**FIGURE 1:**
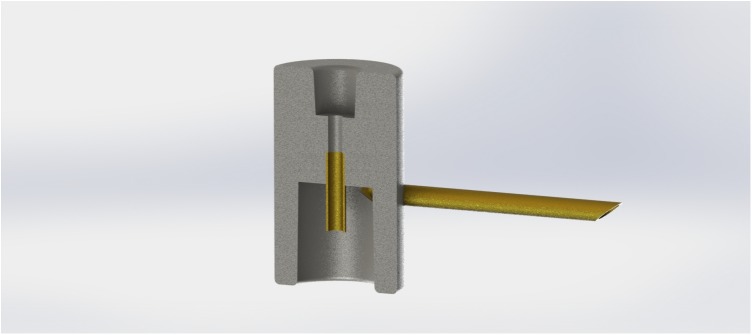
Cross section of a digitally designed cyclone spore collector used in handling cereal rust urediniospores.

**FIGURE 2:**
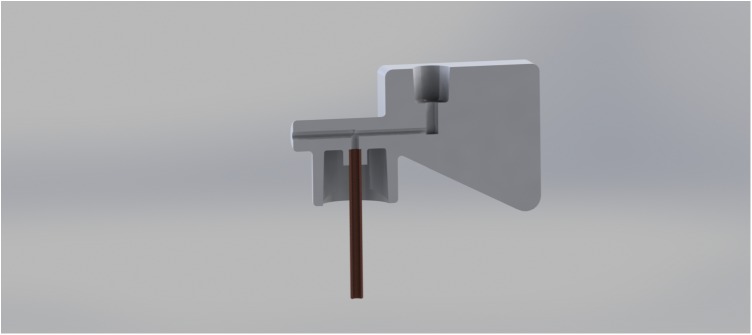
Cross section of a digitally designed inoculator used in handling cereal rust urediniospores.

Based on the descriptions by Browder (1971), devices were digitally designed with Solidworks^®^ software (Figure 1, 2) and manufactured in nylon using an EOSINT P 385 thermoplastics laser sintering system. PA 2200, a polyamide 12 based powder which serves a variety of applications^1^, was used in the sintering process.

**FIGURE 3:**
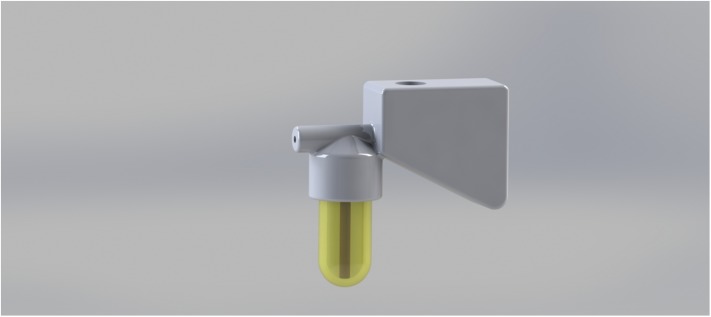
Design of a complete inoculator fitted with a copper tube and containing a gelatin capsule.

**FIGURE 4:**
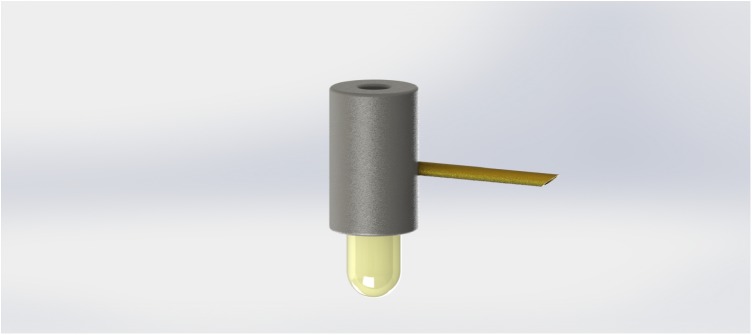
Design of a complete collector fitted with a copper tube and containing a gelatin capsule.

Once the body of a device was manufactured, excess powder was removed from flow channels by compressed air. For each device, a copper or aluminum tube fitted afterward allowed for either the controlled collection of fungal spores from rust pustules, or was used as a siphon tube connecting the inoculator body with the spore suspension in the capsule (Figure 3, 4).

### Device Testing

#### Spray Time

The spray delivery time of AM inoculators and traditionally manufactured metal inoculators was compared. For this purpose, the time (seconds) to discharge 0.8 ml carrier Soltrol 130^®^ isoparaffinic oil at a 25 kPa pressure setting, using a Vacuubrand^®^ pump (model MZ2), was recorded for 10 inoculators of each type. The experiment was designed to include two sprays for each inoculator and an independent but similar repetition, using different sets of inoculators.

#### Spore Deposition

The number of urediniospores deposited per centimeter square was determined by spraying a suspension of 0.4 mg spores per ml Soltrol 130^®^ isoparaffinic oil onto microscope slides. To mirror the freehand spray pattern of an actual seedling inoculation protocol, the glass slides, covered with a thin layer of Plantex^®^, were vertically positioned on a rotating platform in the inoculation booth. To allow for a quantifiable number of spores per unit area, the adhesive side of each slide passed only once through the spray cloud. Three slides were sprayed with each of two AM and two metal inoculators, respectively. The number of urediniospores applied was counted, using a stereo microscope, on two 1-cm^2^ squares drawn earlier on the lower side of each slide. Identical experiments were conducted for urediniospore deposition of *Puccinia triticina* (*Pt*, causal agent of wheat leaf rust) and *P. graminis* f. sp. *tritici* (*Pgt*, causal agent of wheat stem rust).

#### Handling Single-Pustule Isolates

The efficiency of collectors and atomizers in handling urediniospores from individual leaf or stem rust uredinia (pustules) was tested. Following collection of spores from a single uredinium using an AM cyclone collector connected to a vacuum pump, 0.3 ml Soltrol 130^®^ isoparaffinic oil was added to the capsule and sprayed onto one pot of 7-day-old Morocco wheat seedlings. Morocco, which is susceptible to both leaf and stem rust, was grown in Mikskaar Professional Potting Soil 70 (distributed by Hygrotech, Pretoria, South Africa) in 10-cm diameter plastic pots. This process was repeated using an additional three different collectors and atomizers for each of the rust pathogens and the entire experiment was repeated. The number of leaves studied per pot ranged from 31 to 51, and from 39 to 49, in the *Pt* and *Pgt* single-pustule tests, respectively.

To determine the level of infection on the number of plants that would constitute a regular differential set as used in phenotypic race analysis, four pots, each containing five clumps of Morocco, were inoculated with spores collected from a single pustule suspended in 0.8 ml Soltrol 130^®^ isoparaffinic oil. This procedure was conducted for both rust pathogens. In total, 123 seedling leaves were evaluated for *Pt* and 113 leaves for *Pgt* infection with spores collected from a single uredinium, respectively.

#### Handling Bulk Spore Collections

Infection levels on primary leaves of Morocco seedlings were investigated in a spore multiplication procedure. Using urediniospores collected in bulk and Soltrol 130^®^ isoparaffinic oil, 0.8 ml inoculum of each pathogen was applied to four pots of Morocco seedlings. The spore concentration was 1 mg spores / ml oil for *Pt* and 5 mg / ml for *Pgt*. Pots were placed on a rotating disk in an enclosed inoculation booth and fully expanded primary leaves were spray-inoculated by connecting the Vacuubrand^®^ pump, registering a pressure of 25 kPa during operation, to the atomizer. For each pathogen this treatment was replicated using different atomizers.

In all infection experiments the inoculation booth was flushed with water for 30 s between sprays and standard protocols for cleaning, post-inoculation drying of seedlings, dew chamber incubation and maintenance in the greenhouse were applied (Pretorius et al., 2012). Since the response of wheat lines in routine screening or race typing experiments is determined on a qualitative infection type, the number of infected leaves (infection frequency) and minimum number of uredinia on any leaf were determined in the experiments described above.

#### Sterilization

Sterilization tests for AM collection and inoculation devices were conducted with *Pt* and *Pgt*, using metal devices as control treatments. Collectors and inoculators of each type, cleaned according to the regular protocol for metal devices at the UFS rust laboratory, were used to initiate experiments.

For each of *Pt* and *Pgt*, six AM and six metal collectors were exposed to urediniospores as would be experienced during routine spore handling operations. Three collectors of each type and for each pathogen were then submerged in acetone for 60 s followed by a 2 h heat treatment at 75°C in an oven. The remaining collectors were considered as unsterile controls. Clean gelatin capsules were attached to each collector and a vacuum generated in the absence of any urediniospores. Each capsule was filled with 0.3 ml carrier oil which was sprayed, using a clean inoculator, onto one pot of Morocco seedlings.

In a mock inoculation treatment, six inoculators of each type and for each pathogen were used to spray 0.8 ml of a 0.5 mg urediniospore per ml oil suspension. Similar to the collectors, half of the inoculators were then submerged in acetone for 60 s and heat-treated at 75°C for 2 h. Using the treated and untreated inoculators, 0.3 ml clean carrier oil was sprayed per device onto one pot of Morocco seedlings. The infection frequency and number of pustules per leaf were recorded.

#### Statistical Analysis

Data for spray delivery time, urediniospore deposition, and infection frequency of untreated AM and metal devices were analyzed for variance using NCSS v.8 statistical software. Since analysis of variance did not indicate any factor as a significant source of variation, data from duplicate experiments was pooled and no further mean separation tests applied.

## Results

### Spray Time

According to analysis of variance neither device type (*F* = 1.15, *P* = 0.289), individual inoculator (*F* = 1.61, *P* = 0.139), replicates (*F* = 0.02, *P* = 0.886) nor experiments (*F* = 0.4, *P* = 0.528) significantly influenced results (Table 1). The mean time for AM inoculators to deliver 0.8 ml carrier oil was 11.1 s as opposed to 10.6 s for the metal devices. Variable delivery times occurred for both device types with 8.1 to 15 s being recorded over replicates and experiments for AM inoculators and 7.1 to 14.9 s for metal inoculators (Table 1).

**Table 1:** Spray delivery time of and urediniospore deposition by inoculation devices produced by Additive Manufacturing (AM) in comparison with traditionally manufactured metal inoculators.

Criterion		Device type	Variable^1^	Range
**Spray time**			**Mean time (s)**	

		AM	11.1	8.1 – 15.0
		Metal	10.6	7.1 – 14.9

**Spore deposition**	**Rust species^2^**		**Mean no. spores per cm^2^**	

	*Pt*	AM	102	93 – 115
		Metal	108	70 – 137
	*Pgt*	AM	111	80 – 151
		Metal	113	69 – 148


*^1^According to analysis of variance for both spray time and spore deposition experiments no factors were significant sources of variation; ^*2*^*Pt*, *Puccinia triticina* and *Pgt*, *P. graminis* f. sp. tritici.*

### Spore Deposition

No significant variation was revealed for rust species (*F* = 1.48, *P* = 0.234), device type (*F* = 0.36, *P* = 0.779), or replicates (*F* = 0.47, *P* = 0.5) (Table 1). The mean of 102 *Pt* urediniospores deposited per cm^2^ by an AM inoculator was statistically similar to the mean of 108 spores deposited by metal inoculators. Likewise, *Pgt* spore numbers per unit area were closely related for both device types, with 111 spores per cm^2^ for AM atomizers and 113 for metal ones (Table 1). Over replicates and experiments, *Pt* spore numbers ranged from 93 to 115, and from 70 to 137 for AM and metal inoculators, respectively. For *Pgt*, counts ranged from 80 to 151, and 69 to 148 spores per cm^2^ for the respective device types (Table 1).

### Handling Single-Pustule Isolates

**FIGURE 5:**
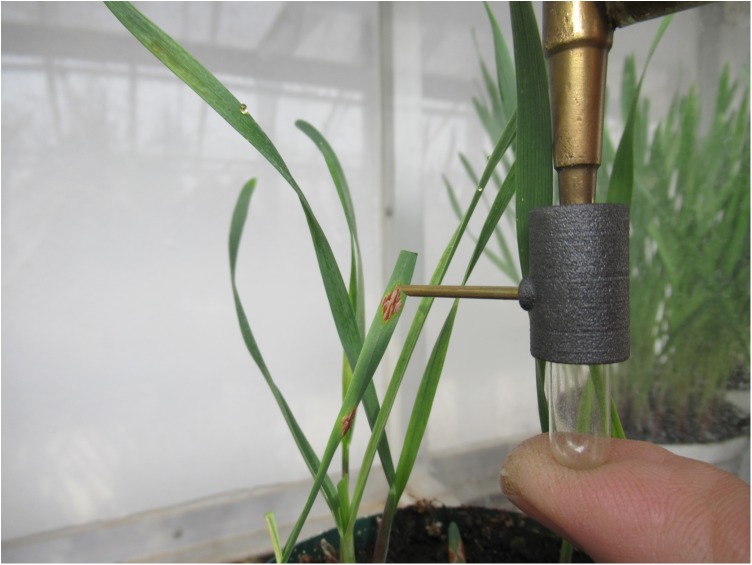
Collection of urediniospores from a single pustule of *Puccinia graminis* f. sp. *tritici* on a wheat leaf using a cyclone spore collector constructed with Additive Manufacturing (AM). The brass T-piece, which connects devices to either a vacuum or pressure pump, is also shown.

The collection of a single rust pustule and the spray cloud discharged from an inoculator are shown in Figure 5, 6. Deposition of urediniospores collected from a single pustule of either *Pt* or *Pgt* (Figure 7) produced satisfactory and repeatable infection levels on wheat seedlings (Table 2). When a single *Pt* pustule suspension of 0.3 ml was applied to one pot of wheat seedlings, a 100% infection frequency and minimum of 10 pustules per leaf were observed. Likewise, the application of 0.3 ml inoculum containing spores from a large, single *Pgt* pustule produced 15 or more pustules per leaf with all plants in a pot being infected.

**FIGURE 6:**
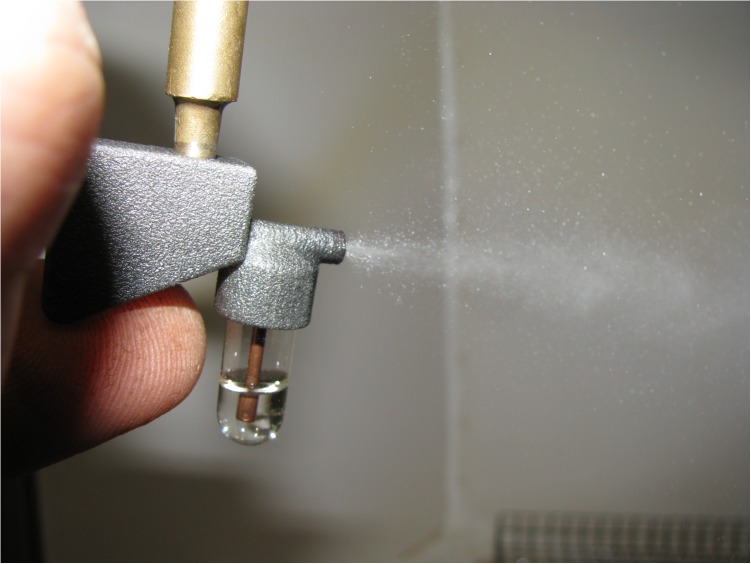
Discharge of light mineral oil by an inoculator constructed with AM. The brass T-piece, which connects devices to either a vacuum or pressure pump, is also shown.

**FIGURE 7:**
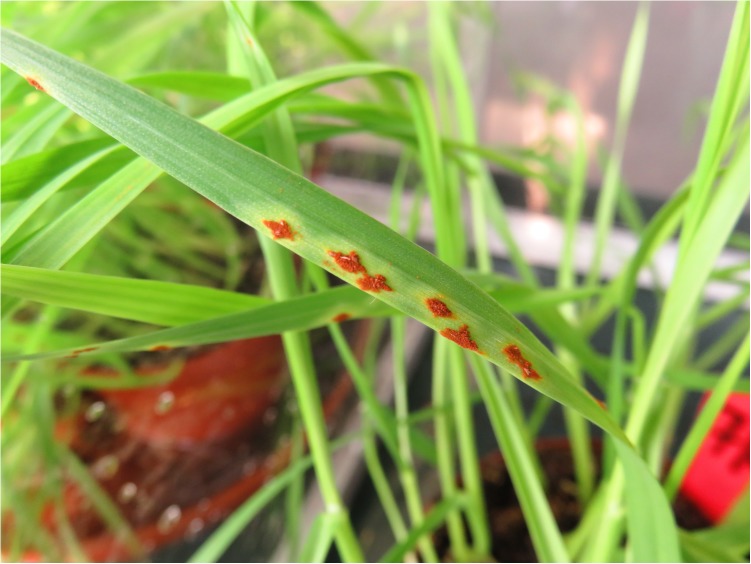
Infection of a primary wheat leaf after inoculation of a differential set of 20 entries with a single pustule of *P. graminis* f. sp. *tritici* using an inoculator produced with AM.

**Table 2:** Efficiency of spore collectors and inoculation devices produced by AM in producing rust infection on wheat seedlings and verification of sterilization procedures in comparison with traditionally manufactured metal inoculators.

Criterion	Rust species^1^	Treatment^2^	Device type	Number of leaves^3^	Infection frequency^4^	Minimum no. pustules per leaf
Rust infection	*Pt*	SPI / pot	AM	248	100	10
	*Pgt*	SPI / pot	AM	268	100	15
	*Pt*	SPI / 4 pots	AM	123	100	4
	*Pgt*	SPI / 4 pots	AM	113	100	5
	*Pt*	Bulk / 4 pots	AM	310	100	29
	*Pgt*	Bulk / 4 pots	AM	364	100	7

**Device sterilization^5^**						**Pustule numbers per leaf**

Collector	*Pt*	Treated	AM	146	0	0
	*Pt*	Control	AM	149	24.6	1
	*Pt*	Treated	Metal	155	0	0
	*Pt*	Control	Metal	144	49.7	1 – 6
	*Pgt*	Treated	AM	147	0	0
	*Pgt*	Control	AM	144	28.6	1 – 2
	*Pgt*	Treated	Metal	149	0	0
	*Pgt*	Control	Metal	149	29.3	1 – 3
Inoculator	*Pt*	Treated	AM	145	0	0
	*Pt*	Control	AM	148	44.0	2 – 3
	*Pt*	Treated	Metal	145	0	0
	*Pt*	Control	Metal	147	34.4	1 – 2
	*Pgt*	Treated	AM	151	0	0
	*Pgt*	Control	AM	144	61.5	2 – 5
	*Pgt*	Treated	Metal	141	0	0
	*Pgt*	Control	Metal	144	45.4	1 – 4


*^1^*Pt*, *Puccinia triticina* and *Pgt*, *P. graminis* f. sp. *tritici*; ^*2*^SPI, single pustule isolate and bulk = bulk isolate; ^*3*^Total number of wheat seedling leaves assessed over replications; ^*4*^Percentage of wheat seedlings showing signs and symptoms of rust infection; ^*5*^Sterilization consisted of submerging devices for 60 s in acetone followed by a 2 h heat treatment at 75°C. Collectors and devices not cleaned after exposure to urediniospores served as control treatments.*

**FIGURE 8:**
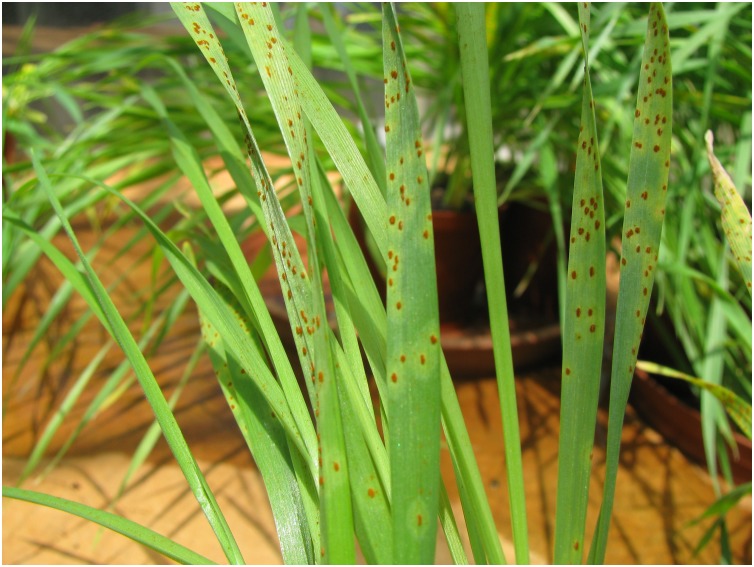
*Puccinia* *triticina* infection obtained with an inoculator produced with AM.

In the simulated differential set distributed as 20 “entries” over four pots and sprayed with single pustules of either *Pt* or *Pgt* suspended in 0.8 ml oil, all seedling leaves were infected by both pathogens. The number of rust pustules was evenly distributed with a minimum of 4 and 5 pustules recorded per leaf for *Pt* and *Pgt*, respectively (Table 2). In general, higher infection levels were obtained with *Pt* (Figure 8).

### Handling Bulk Spore Collections

Application of 0.8 ml *Pt* inoculum at 1 mg spores / ml oil to four pots of Morocco seedlings, as would be used in a spore bulking exercise, resulted in a minimum of 29 pustules on a leaf but more than 40 on others. The stem rust spore multiplication test, using 0.8 ml of a 5 mg / ml suspension, ensued in 7 to 30 pustules per leaf. Similar to previous tests, a 100% leaf and stem rust incidence was obtained (Table 2).

### Sterilization

During validation of the sterilization process, leaf rust infection frequencies of 24.6 and 49.7% were obtained from spores accumulated in clean gelatin capsules attached to unsterile AM and metal collectors, respectively (Table 2). Contamination levels reached 1 to 6 pustules per seedling leaf. For *Pgt*, the infection frequencies resulting from used AM and metal collectors were 28.6 and 29.3%, with 1 to 3 contaminant pustules per leaf. When unsterile inoculators of both types were used to spray Morocco seedlings with clean oil, contamination ranging from 34.4 to 61.5% infected plants was observed amongst the *Pt* and *Pgt* treatments. Number of pustules varied between 1 and 5 per leaf. According to analysis of variance, infection frequency data obtained for unsterile collectors did not differ significantly for rust species (*F* = 0.84, *P* = 0.396) or device type (*F* = 2.08, *P* = 0.2). Similarly, infection data from unsterile inoculators was statistically alike for rust species (*F* = 2.34, *P* = 1.77) and device type (*F* = 1.9, *P* = 0.217). No infection sites were observed on seedlings sprayed with inoculators first submerged in acetone and then dried at 75°C for 2 h (Table 2).

## Discussion

Depending on infrastructure and standard operating procedures, research laboratories employ different systems when inoculating host plants with urediniospores of rust fungi. If spray-inoculation is preferred, spores could be suspended in water, isoparaffinic oil or engineered fluid. Alternatively, dry spores can be allowed to settle on plant surfaces by dusting or directly applied with a spatula or small brush.

Use of a spore-oil suspension, contained in a gelatin capsule and sprayed onto seedling leaves by means of a dedicated atomizer connected to an air pressure source, is favored by many rust laboratories due to ease of use, small amounts of inoculum required and consistency in infection levels. The uniform deposition of inoculum on seedling leaves by these venturi atomizers, which expel the suspension as a solid cone of fine droplets when pressurized air passes through a throat over the end of the fluid delivery tube, was emphasized by Rowell (1985). A further advantage is that the same capsule used for spore collection is used for inoculation after oil has been added. A disadvantage of the system, irrespective of whether conventional or AM devices are used, is that the gelatin capsules liquefy when they come into contact with water. However, 0.5 ml Eppendorf^®^ microcentrifuge tubes fit the dimensions of the AM collector and inoculator and can be used with minimal alteration should fungal spores dispersed in water be a requirement. The AM devices could not be manufactured in a single step operation but required manual assembly of the copper tubes. In addition, for some inoculators it was necessary to clear the small delivery tube from presumably powder deposits. Although the PA 2200 material used in the production process features desirable strength and stiffness properties, chemical and heat resistance and consistent long-term behavior (see footnote 1), the durability of the AM devices needs to be verified after prolonged use.

The availability of a large quantity of collectors and inoculators is useful when many isolates need to be applied in a single session, such as in pathogen race typing experiments. In view of the re-emergence of wheat rusts on several continents (Olivera et al., 2015; Singh et al., 2015; Newcomb et al., 2016; Ali et al., 2017; Olivera Firpo et al., 2017; Lewis et al., 2018) several international laboratories are engaged in multi-isolate experiments where spore collection and inoculum application are done on a regular basis, reflecting the potential value of the equipment.

Despite their advantages, neither the cyclone collectors nor the atomizers are commercially available in South Africa. Local manufacturing by conventional milling from a solid metal block requires a specialized workshop and skilled personnel with consequent time and cost implications. Inoculators developed with AM did not differ significantly from the traditional metal devices in spray time or spore deposition. Using urediniospores from single rust pustules or bulk spore samples, AM devices were consistent in spore collection and infection of wheat seedlings and provide a viable alternative to metal devices originally obtained by means of a subtractive and labor intensive process. Similar to the procedure described by Browder (1971), the operation of a collector or inoculator requires connection to a vacuum or pressure source, respectively. To facilitate these unions, a T-piece manufactured from brass was used in our experiments. The long end of the T-piece connects to the air source by means of rubber tubing, another fits into the tapered friction joint of the apparatus, whereas the third opening is closed manually when either suction or pressure is required.

## Author Contributions

ZP conceptualized the idea. GB, JJ, and JE designed and manufactured the prototypes. WB, GM, and ZP conducted the validation tests.

## Conflict of Interest Statement

The design of the inoculator has been registered in Europe, United States, and South Africa. Neither the employing institutions nor any of the authors hold a patent on the devices described. Should a third party be interested in ordering any number of the devices, the University of the Free State and Central University of Technology will be reimbursed. The authors declare that the research was conducted in the absence of any commercial or financial relationships that could be construed as a potential conflict of interest.

## Footnotes

1 https://www.eos.info/
